# The Role of Cyanoacrylate after Mandibular Third Molar Surgery: A Single Center Study

**DOI:** 10.3390/bioengineering11060569

**Published:** 2024-06-05

**Authors:** Stefano Parrini, Giulia Arzente, Elena Bartali, Glauco Chisci

**Affiliations:** Oral Surgery Postgraduate School, Dentistry and Dental Prosthodontics, Department of Medical Biotechnologies, University of Siena, 53100 Siena, Italy

**Keywords:** suture, infection, cyanoacrylate, silk, surgery, third molar, extraction, sutureless, bacterial adhesion

## Abstract

Background: The management of the surgical wound of partially impacted mandibular third molar surgery has a great impact on recovery as well as on food impact retention. The present study used clinical parameters and health-related quality of life (HRQL) to compare outcomes of cyanoacrylate application versus traditional suture of third molar impaction surgery. Methods: This was a retrospective observational study of subjects scheduled for outpatient third molar surgery. Each participant signed an informed consent agreement. Inclusion criteria were as follows: presence of at least one partially impacted mandibular third molar, confirmed with a preoperative panoramic radiograph. Exclusion criteria were the following: smoking, diagnosed diabetes mellitus. Between June 2020 and September 2023, a total of 78 patients of mean age 31.14 years old (range 21–40 years, standard deviation 9.14), were included in this study—38 patients were male, 40 patients were female. A group of patients received traditional silk suture (G1 = 41 patients), while the second group (G2 = 37 patients) received hemostasis performed with fibrin sponge and, after complete soaking of the sponge, the application of cyanoacrylate gel on the blood clot and suture with one 2/0 stitch in order for recovery for secondary closure. The following parameters were measured: HRQL, average pain (AP), maximum pain (MP), complication score (CS), facial swelling (FS), and erythema. Results: For HRQL parameters, oral disability was found to be significantly higher in G1 while AP was significantly higher in G2 (*p* < 0.05). AP was higher in G2 (*p* = 0.0098), as well as MP (*p* = 0.001). No differences were found with regards to CS (*p* = 0.0759). FS and erythema were higher in G1 (*p* < 0.0001 for facial swelling, and *p* = 0.0001 for erythema). Conclusions: on the basis of this study, the use of cyanoacrylate after mandibular third molar surgery appears to be useful in order to reduce postoperative oral disability, facial swelling, and erythema after tooth extraction, with increased average and medium pain: clinicians may consider its use in selected cases.

## 1. Introduction

Mandibular third molar removal is a common intervention in oral and maxillofacial surgery. Although there may be many indications for the extraction of the mandibular third molar, such as orthodontic indication or contact with jaw cyst, the most common cause of third molar extraction remains pericoronaritis, as an infection and inflammation of the pericoronal tissues around the third molar [1]. At the end of surgery, the hemostasis plays an important role for preventing blood clot loss and promoting healing after the extraction: this aspect is important for both outpatients and hospitalized patients. Mandibular third molar extraction represents an operation that leaves an alveolar socket huge in dimensions, both for the erupted and partially erupted teeth. While this aspect is less important in the fully impacted third molar for the partially impacted and completely erupted teeth, this may lead to an unpleasant recovery [2]. The management of the surgical wound of partially impacted mandibular third molar surgery has a great impact on its recovery as well as the food impact retention. The space distal to the second molar may facilitate dehiscence and food impact, with poor recovery for secondary intention healing and may promote microbial retention and infection due to the food impact. Carrasco-Labra et al. [3] in their review in 2012 reported no difference between primary and secondary closure in third molar surgery. However, in cases of correct healing Ma et al. [4] in 2019 reported better recovery for the secondary intention closure compared to the primary intention closure. More recently Azab et al. [5] in their review reported in 2022 small advantages at day 1 and day 3 in terms of swelling for the secondary closure compared to the primary closure after third molar surgery. While the primary closure may allow fewer complications after third molar surgery, it is associated with worse postoperative recovery; on the other hand, the secondary recovery leads to prolonged healing with better postoperative recovery and symptoms, but with a possible infection due to food retention. On the basis of the choice between primary or secondary closure, the concept of suture and hemostasis plays an important role, both for very complex extractions and easy flapless extractions. Nevertheless, many factors may influence recovery after third molar surgery, such as operation time, difficulty, preoperative inflammation, and surgical experience [6,7,8,9]. Among the aspects that an oral surgeon may control in order to reduce postoperative morbidity, the suture technique was thoroughly investigated: the suture-less technique and one-suture technique proved to influence the recovery after mandibular third molar surgery [10,11,12]. Recently, the use of cyanoacrylate gel was introduced in oral surgery: Cyanoacrylate is a type of adhesive that is known for its fast-drying and strong-bonding properties. It is commonly referred to as “super glue” or “instant glue” because it forms a bond quickly when exposed to moisture in the air. Cyanoacrylate adhesives are used in various applications, including bonding metals, plastics, ceramics, rubber, and more [13].

Cyanoacrylate is commonly used in medicine and surgery as a medical adhesive or tissue adhesive. It is used for wound closure instead of traditional sutures or staples in certain situations. Cyanoacrylate adhesives can create a barrier that protects the wound from bacteria and foreign particles, helping to accelerate the healing process. In surgical procedures, cyanoacrylate can be used to seal or bond tissues or to stop bleeding in delicate areas. Additionally, it is sometimes used in dermatology for skin conditions or wound treatment. Its quick bonding properties and ability to create a strong seal make it a valuable tool in medical settings. Several authors reported the first use of cyanoacrylate gel in oral surgery for treatment of mandibular venous malformation and dental extractions in patients with hereditary bleeding disorders [14,15].

Although bleeding is not a common complication in dentoalveolar surgery in healthy patients, the use of cyanoacrylate as an alternative to suture after mandibular third molar surgery has been considered by many investigators. The present study used clinical parameters and health-related quality of life (HRQL) to compare outcomes of cyanoacrylate application versus traditional suture of third molar impaction surgery. 

## 2. Materials and Methods

### 2.1. Patients

This study enrolled patients previously visited and scheduled for outpatient third molar surgery at the Oral Surgery Postgraduate School, Dentistry and Dental Prosthodontics, Department of Medical Biotechnologies, University of Siena between June 2020 and September 2023. Each participant signed an informed consent agreement.

### 2.2. Inclusion and Exclusion Criteria

For all cases acute pericoronitis was the indication for surgery. Inclusion criteria were the following: the IIB classification of Pell and Gregory, partially impacted mesio-angulated mandibular third molar, confirmed with a preoperative panoramic radiograph [16], and preoperative pain.

Exclusion criteria were as follows: impaired communicative or cognitive disease, diagnosed diabetes mellitus, oral surgical intervention within 30 days before data collection, third molars related to mandibular neoformations. The observational retrospective design did not require the approval of an ethics committee, as per the Italian legislation on clinical investigations at the time of the study. Nevertheless, the investigation was carried out following the rules of the Declaration of Helsinki of 1975, revised in 2013, and performed according to the principles of the ICH Good Clinical Practice.

A total of 78 patients of mean age 31.14 years old (range 21–40 years, standard deviation 9.14) were included in this retrospective study: 38 patients were male, 40 patients were female. A group of 41 patients received traditional silk suture (G1), while the second group of 37 patients (G2) received hemostasis performed with fibrin sponge and, after the complete soaking of the sponge, application of cyanoacrylate gel on the blood clot and suture with one 2/0 stitch in order to recover for secondary closure. Demographic data of the patients are reported in Table 1.

**Figure 1:**
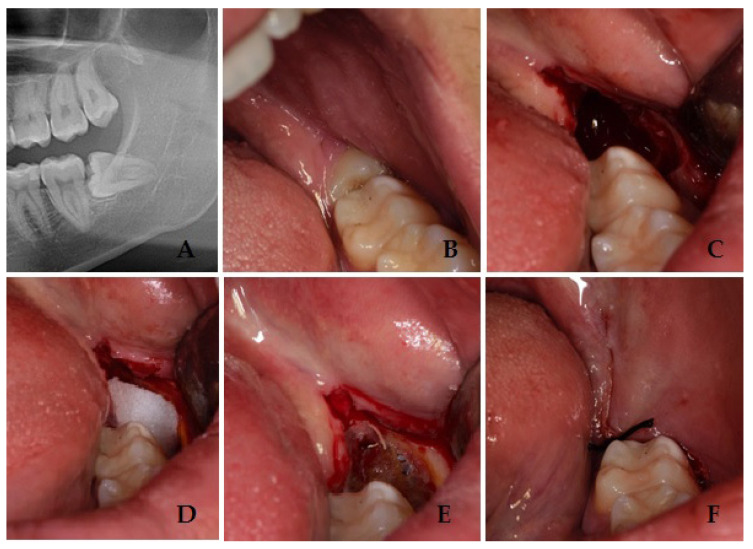
Panoramic radiograph that shows the impacted third molar (**A**); preoperative figure with third molar (**B**); intraoperative figure after tooth removal and blood clot (**C**); intraoperative figure with fibrin sponge (**D**); intraoperative figure with cyanoacrylate gel after polymerization (**E**); postoperative figure after suture (**F**).

Both groups received oral hygiene in the month before surgery and were trained for correct home oral hygiene. All interventions were performed by the same surgeon with experience of more than 20 years in oral surgery (S.P.). After local anesthesia with articaine 1:100.000, an incision with a scalpel was performed distally to the second mandibular molar with vestibular release incision. At this point a full-thickness mucoperiosteal flap was elevated with periosteal elevator. Around the mandibular third molar crown osteotomy, procedure was then performed with bur, the tooth was then dislocated with a straight lever and completely removed with pliers. Accurate alveolar revision was then performed, and a saline lavage was conducted inside the alveolus. After the tooth extraction, G1 patients received hemostasis performed with fibrin sponge and suture with one 2/0 stitch, in order to recover for secondary closure. G2 patients instead received hemostasis performed with fibrin sponge and, after the complete soaking of the sponge, the application of cyanoacrylate gel (Glubran 2, GEM, Italy) on the blood clot and suture with one 2/0 stitch in order to recover for secondary closure (Figure 1).

Patients were educated not to brush on the surgical site and to avoid rinses with mouthwashes. All patients underwent two different postsurgical clinical examinations, the first one 7 days after surgery, occurring at the time of suture removal, and the second one 14 days after the surgery. On the 7th day there was an interim evaluation, while the 14th day evaluation was an important reference as most of the symptoms after third molar surgery had disappeared.

The evaluation of postoperative recovery and oral disability was performed with the following parameters 14 days after surgery: health-related quality of life (HRQL); average pain (AP); maximum pain (MP); complications score (CS); facial swelling (FS); erythema.

### 2.3. Health-Related Quality of Life

Each patient received an “HRQL diary”, including 14 copies of the daily data collection page. Measuring HRQL can provide valuable insights into how a person’s health status affects their daily life, overall satisfaction, and functional ability. It also helps healthcare providers and researchers to understand the broader impact of diseases and treatments beyond clinical outcomes, guiding decision-making processes and improving patient-centered care. Patients were taught to complete 1 page each evening for the 14-days beginning at the first day of surgery. After mandibular third molar surgery, all patients received standardized clinical postoperative follow-up checks, and oral disability after the intervention was measured with standardized parameters of HRQL diaries, measuring oral function, general activities, and other functions. General activity is a measure of the ability to follow daily routine activities, sleeping, recreation, and social interaction. Further, symptoms like bleeding, swelling, bruising, food impaction in the surgical sites, the presence of bad taste or bad breath, and nausea were recorded.

### 2.4. Pain

Pain measures were present on each page of the diary and included average pain (AP) and maximum pain (MP) with a 0–10 score with measurements performed with a visual analogue scale.

### 2.5. Complications Score

The possible occurrence of postsurgical complications was clinically assessed by S.P. and measured as a score (CS, 0–10 score). Severe complications included the following: (1) evidence of dry socket/alveolar osteitis; (2) occurrence of mandibular fracture; (3) inferior alveolar nerve and lingual nerve lesions. Milder complications at 7 and 14 days included soft tissue infection, evocated pain, bone resorption, and loss of stitches.

### 2.6. Facial Swelling and Erythema

Facial parameters were evaluated before surgery and after 7 days and 14 days from surgery; clinical evidence was assessed for erythema (0–10 score). The evaluation of postoperative FS was measured through specific measurements: the first measurement was detected taking as reference the distance between the mandibular angle and the wing of the nose; the second measurement was performed taking as reference the space between the mandibular angle and the labial commissure angle. The mean value of these measurements resulted in the final value. The increased measurement at day 7 and day 14 was used as the score.

### 2.7. Statistical Analysis

The following variables were evaluated for the two groups (G1 control group; G2 test group): health-related quality of life (HRQL); average pain (AP); maximum pain (MP); complications score (CS); facial swelling (FS); erythema. All variables were tested for normal distribution (D’Agostino–Pearson test). Differences between the groups were evaluated using the Mann–Whitney rank sum test (continuous normally distributed data). *p* values < 0.05 were considered statistically significant. The software MedCalc version 9.5.2.0 (MedCalcSoftware, Mariakerke, Belgium) was used for statistical analysis.

## 3. Results

**Figure 2:**
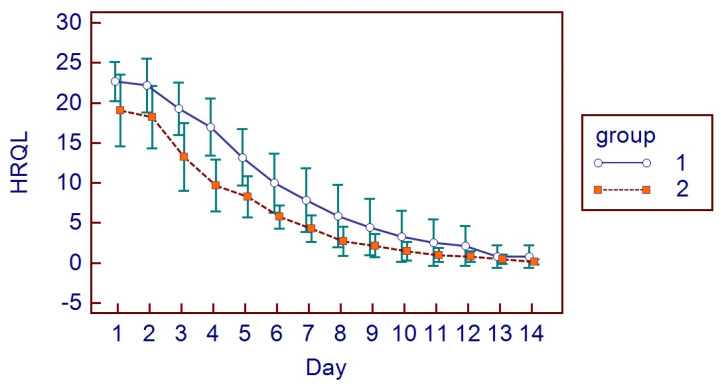
HRQL parameters.

**Figure 3:**
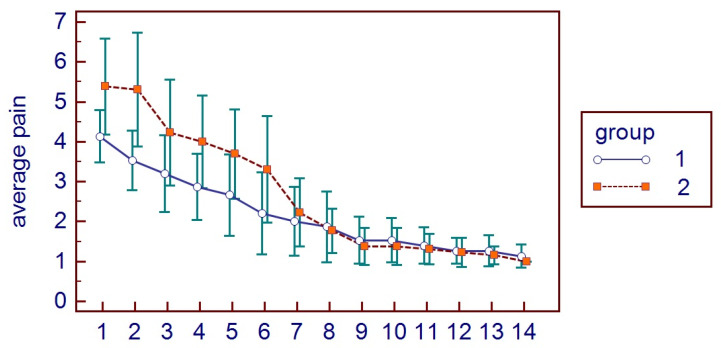
Average pain parameters in the two groups.

**Figure 4:**
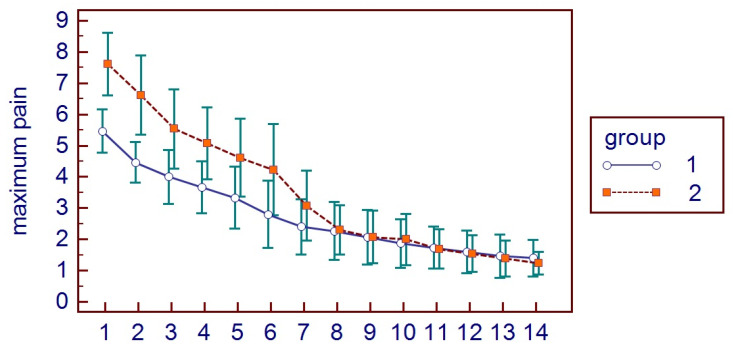
Maximum pain parameters in the two groups.

**Figure 5:**
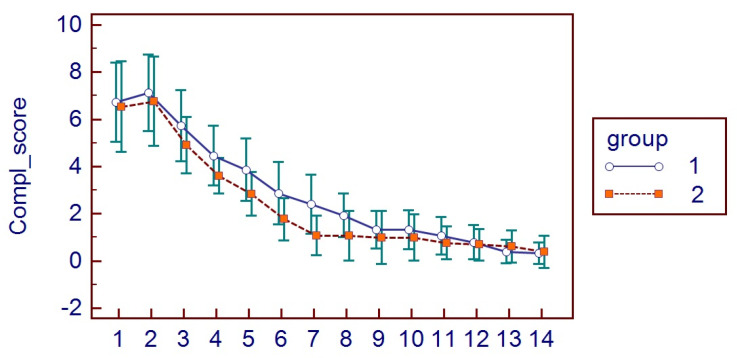
Complication score parameters in the two groups.

**Figure 6:**
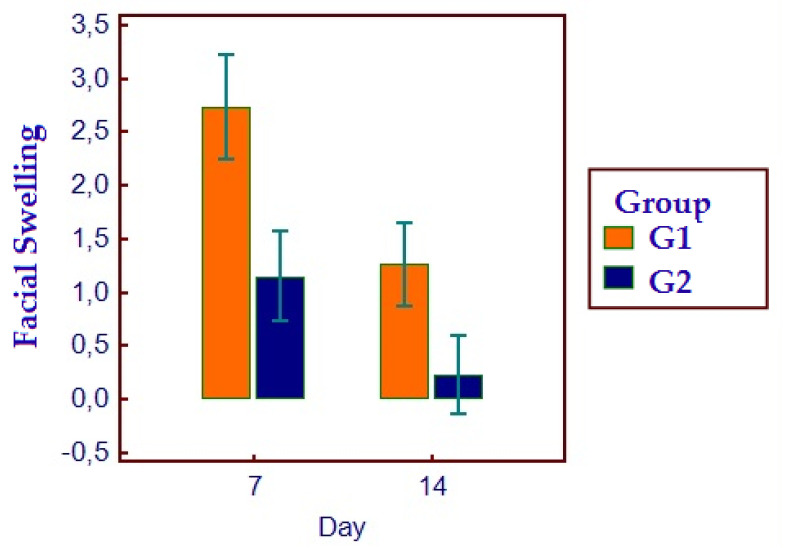
Facial swelling parameters in the two groups.

**Figure 7:**
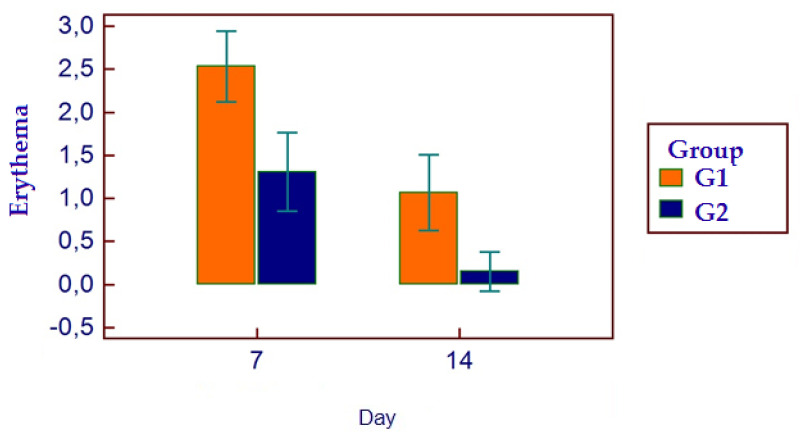
Erythema parameters in the two groups.

All patients presented themselves at the two post-surgical visits and reported all the HRQL diaries. No hemorrhagic complications were reported either in G1 or G2. No postoperative infections or alveolar osteitis were reported in both groups, nor other complications. For HRQL parameters, oral disability was found to be significantly higher in G1 while AP was significantly higher in G2 (*p* < 0.05) (Figure 2). AP was higher in G2 (*p* = 0.0098) (Figure 3), as well as MP (*p* = 0.001) (Figure 4). No differences were found with regards to CS (*p* = 0.0759) (Figure 5). FS and erythema were higher in G1 (*p* < 0.0001 for facial swelling, and *p* = 0.0001 for erythema) (Figure 6 and Figure 7).Mean values of variables are reported in Table 2.

## 4. Discussion

The management of postoperative morbidity after third molar extraction is a matter of great interest in oral and maxillofacial surgery. Health related quality of life is a common item to evaluate postoperative discomfort after mandibular third molar surgery [17]. Health-related quality of life (HRQL) is a multidimensional concept that encompasses an individual’s physical, mental, emotional, and social well-being in relation to health status. It represents an individual’s subjective perception of the impact of an illness, injury, or treatment on their overall quality of life. HRQL assessments often evaluate aspects such as physical functioning, emotional well-being, social functioning, and symptoms related to health conditions. For this reason, we routinely evaluate the outcome of third molar surgery with HRQL parameters and our results are in line with international literature in terms of worsening of quality of life after third molar extraction [18,19,20].

With regards to the role of suture after third molar surgery, Mahat et al. [21] reported good results with a sutureless technique in third molar surgery with reduced swelling in the sutureless group compared to the suture group: this concept still needs a systematic review as this technique is reported to have divergence among surgeons. This matter has been discussed many times in literature: primary and secondary closure represent a challenging matter for many surgeons in order to influence the recovery after third molar extraction and to obtain a better surgical outcome.

The introduction of cyanoacrylate in third molar surgery has an endearing history: Møller [22] was the first author in 1988 to use the sealant in the alveolus after 61 third molar extractions with no differences in the first weeks. Recently, the use of fibrin and clot sealants and the possibility to avoid sutures gathered interest among surgeons. Marco de Lucas et al. [23] reported good results in the treatment of a life-threatening pseudoaneurysm after a dental extraction. Ghoreishian et al. [24] in their comparative study reported that postoperative bleeding with the cyanoacrylate method was less compared to suturing on the first and second days after the extraction, while there was no significant difference in the severity of pain between the two methods. Joshi et al. [25] in 2011 suggested both cyanoacrylate and suturing in wound closure were similar in the entity of pain, but patients with cyanoacrylate showed better hemostasis: our experience on 78 patients confirmed the enhanced hemostasis in the G2 cyanoacrylate group. On the other hand, in this study the G2 cyanoacrylate group experienced significantly more pain compared to the traditional suture group. With regards to postoperative infection, cyanoacrylate showed the same results of suture, and no dry socket or alveolar osteitis were reported.

With regards to the management of postoperative hemorrhagic complications, some authors used selective transarterial embolization in order to treat the emergence after third molar extraction or to enhance coagulation after venous mandibular neoformation surgery [26,27,28,29]. Gogulanathan et al. [30] in their interesting randomized slit-mouth clinical trial reported superior results after third molar extraction in the group with fibrin sealant compared to traditional suture, with superior values of postoperative wound closure and mouth opening and reduced values in duration to achieve hemostasis and pain score. The results of the present study referring to pain contrast with Gogulanathan et al., as the G2 cyanoacrylate group experienced significantly more pain compared to the traditional suture group: however, the G2 cyanoacrylate group experienced better results in terms of HRQL. The results of the present study, in terms of complication score referring to wound healing, report no statistically difference among the two groups, underlying the variability present in literature. With regards to facial swelling, the results of the present paper support the previous results by Gogulanathan et al. [30].

This study is the first research article to use the HRQL parameters to evaluate postoperative recovery with or without cyanoacrylate gel. Oladega and Adeyemo [31] in their randomized controlled trial between cyanoacrylate and silk suture after mandibular third molar surgery reported that no significant difference was observed in the mean postoperative pain, swelling, trismus, wound dehiscence, and infection between the two groups, with an increased bleeding in the traditional silk suture group compared to the test group. The bleeding after third molar surgery and the management of alveolar socket are great matters of interest in third molar surgery, and evaluations of preoperative tooth pathology and postoperative complication are matters that should be investigated further [32]. In a previous study, Parrini et al. reported differences in plaque retention with PTFE versus silk suture, with an increased plaque retention in silk sutures [33].

A recent systematic review that evaluated cyanoacrylate versus traditional suture reported that both techniques were found to be effective in terms of wound closure, proposing cyanoacrylate as an effective resource that should be investigated in future research. Nevertheless, few papers exist in the literature on cyanoacrylate and they lack comparative results of its outcomes and effects [34]. Santos et al. [35] in their systematic review suggested the use of cyanoacrylate instead of silk suture. The results of our paper add a little knowledge in this context. In the present article we reported a positive outcome for cyanoacrylate gel and single suture after partially impacted mandibular third molar extraction: we conducted a retrospective comparative study with clinical and HRQL parameters. The main limitations and biases of this study are surely the retrospective nature and the lack of randomization: this may have lead to confounding effects that we treated with inclusion and exclusion criteria. Further, the use of a questionnaire for patients could introduce subjective responses but careful interpretation and statistical analysis are required. Finally, in this study the split mouth was not performed, but we used a single suture over the cyanoacrylate gel in order to stabilize the flap. We advocate future studies with larger, multicentric studies to validate the findings across broader populations and we suggest randomizing participants in future studies to minimize bias and better control of confounding variables.

The use of a cyanoacrylate gel under the suture could ease plaque retention: this is an aspect that should be investigated somehow in future research, as the possibility to reduce plaque retention on suture after mandibular third molar surgery represents a matter of concern in order to reduce postoperative complications. Further, more research of cyanoacrylate gel in patients undergoing third molar surgery with antiplatelet therapy or anticoagulant drugs should be addressed. On the basis of our research and experience, we suggest the use of cyanoacrylate in cases of patients with previous bleeding experience in dentoalveolar surgery and simple extractions.

## 5. Conclusions

We found the use of cyanoacrylate to be superior to silk suture with regards to the erythema, facial swelling, and the health related quality of life parameters after mandibular third molar extractions: on the other hand, we found higher values of average pain and maximum pain in the cyanoacrylate group compared to the traditional silk suture group. On the basis of our study, cyanoacrylate does not represent an alternative to traditional suture but a viable option in cases, on the basis of surgeon experience.

## Figures and Tables

**Table 1:** Patient demographic data of G1 and G2.

Patient Demographic Data	Group1	Group2	SMD (Standardized Mean Difference)
Age (mean (SD))	30.53 (±9.77)	31.85 (±10.51)	1.32
Sex (Female/Male)	22/18	17/20	5/2
Surgical site (Right/Left)	19/22	21/16	2/6

**Table 2:** All data are reported as mean values ± (SD).

	G1 Mean ± (SD)	G2 Mean ± (SD)
HRQL	9.43 ± (9.49)	6.23 ± (7.42)
CS	2.88 ± (3)	2.36 ± (2.77)
MP	2.75 ± (1.86)	3.5 ± (2.57)
AP	2.19 ± (1.57)	2.67 ± (2.11)
erythema	1.70 ± (1.08)	0.73 ± (0.82)
FS	2 ± (1.08)	0.69 ± (0.78)

## Data Availability

The original contributions presented in the study are included in the article, further inquiries can be directed to the corresponding author.
